# Unveiling a Large Pelvic Mass in a Middle-Aged Woman: The Consequences of Neglected Gynecological Care

**DOI:** 10.7759/cureus.75789

**Published:** 2024-12-16

**Authors:** Todd R Anderson, Emily J Carletto, Bailey Burns, Augustina Addison

**Affiliations:** 1 College of Medicine, Campbell University School of Osteopathic Medicine, Lillington, USA; 2 Obstetrics and Gynecology, Cape Fear Valley Health, Fayetteville, USA

**Keywords:** benign gynecological surgery, gynecological surgery, gynecology-oncology, mucinous ovarian tumor, obstetrics and gynecology (ob-gyn), onco-gynecology, ovarian tumor

## Abstract

Pelvic masses in women can originate from both gynecological and non-gynecological sources, necessitating careful evaluation to ensure appropriate treatment. Gynecological masses can range from functional ovarian cysts and tubo-ovarian abscesses to malignant and benign tumors. This case report presents a mucinous borderline ovarian tumor (BOT), a rare type of ovarian neoplasm. This case highlights a 48-year-old G6P6006 White female with a history of asthma, presenting to the emergency department with a 10-day history of constipation and a month-long history of abdominal distention, which caused shortness of breath when supine. The patient had no recent primary care or gynecological follow-up. Physical examination revealed abdominal distension with absent bowel sounds and multiple hemangiomas, but no peritoneal signs. Initial laboratory studies were within normal limits, except for an elevated cancer antigen 19.9 (CA 19.9) level of 736.5. Imaging studies, including ultrasound and CT, identified a large complex adnexal mass measuring 28 x 21.5 x 15.4 cm. Given the suspicion of a gynecologic malignancy, the patient underwent a total abdominal hysterectomy with bilateral oophorosalpingectomy and an appendectomy. The mass, which was completely excised, was identified as a mucinous BOT measuring 32 cm at its largest diameter. Intraoperative pathology and peritoneal washings were negative for malignancy. Postoperatively, the patient met all recovery milestones and was discharged on day three, with a successful follow-up for staple removal. This case emphasizes the importance of timely access to healthcare in managing large ovarian tumors and potentially preventing malignant transformation. Improved access to healthcare services, particularly through insurance coverage, can enhance early detection and allow for comprehensive cancer care, thus improving survival outcomes. The case also highlights the lack of routine screening for many gynecological tumors, underscoring the need for improved healthcare access and awareness, which could facilitate earlier intervention and better patient outcomes. Early diagnosis and management of mucinous BOTs can significantly improve prognosis. Addressing healthcare access barriers remains crucial in reducing the risk of late-stage presentations and ensuring effective treatment for women with gynecological malignancies.

## Introduction

Pelvic masses are not exclusively seen in women, but for women, the origin of the mass can arise from gynecological or non-gynecological organs. Careful evaluation of the mass must be elucidated to treat the mass properly. The origin of a gynecologic mass can include functional ovarian cysts, tubo-ovarian abscesses, and malignant or benign tumors [1]. The gynecologic mass of interest is the mucinous ovarian tumor. These tumors can range from benign cystadenomas and borderline ovarian tumors (BOTs) to malignant adenocarcinomas [2]. Ovarian mucinous tumors comprise 10-15% of all diagnosed primary ovarian tumors [3]. Within this pathology, it is estimated that nearly 80% of these masses consist of benign cystadenomas and BOTs [4]. BOTs are characterized by atypical epithelial proliferation without invasion of the stroma [5,6]. This lesion is most prevalent in women younger than 45 [3]. In addition, South Asian and White women are disproportionately affected by tumors with this histology [7]. The clinical manifestation of BOTs is largely mass-dependent, with symptoms ranging from fatigue, bloating, and abdominopelvic pain, and mass-dependent symptoms consisting of constipation and urinary retention [8]. Mucinous borderline tumors are the least common type of tumor, composing one-third of cases of BOTs [9]. The average diameter size of a mucinous BOT is 20 cm [10]. Unfortunately, there is no known screening tool for clinicians to utilize in the early detection of this tumor, leading to high mortality rates with instances of malignant adenocarcinomas [11]. As for mucinous BOTs, the five-year survival rate approaches more than 90% if detected early [12]. Due to the demographics of this disease, treatment for BOTs must consider fertility as a large portion of the population of women is still in their childbearing years [13]. Treatment of BOTs includes fertility-sparing surgery and non-fertility-sparing surgery including total hysterectomies.

## Case presentation

This case presents a G6P6006 48-year-old White female with a past medical history of asthma who presents to the emergency department with constipation for the last 10 days and abdominal distention for the last month. The abdominal swelling is causing shortness of breath when the patient lies down. The patient reported not having a primary care doctor, and her last gynecology appointment was over a decade ago. On initial assessment, the patient's vital signs were within normal limits. Upon physical exam, the abdomen was distended with absent bowel sounds in all four quadrants with nonperitoneal signs. In addition, multiple hemangiomas were noted. The patient did not exhibit asterixis. Labs were gathered from the patient and included a complete blood count (CBC), comprehensive metabolic panel (CMP), and lipase. All lab results were within normal limits. In addition, a urine analysis was performed and demonstrated 2+ leukocytes and 2+ proteins (Table 1). A pregnancy test was obtained, which resulted as negative. A point-of-care ultrasound (POCUS) was performed and revealed a large septated adnexal mass with papillary projections measuring and free fluid within the abdomen.

**Table 1:** Results of urine analysis, demonstrating 2+ for leukocyte esterase and 2+ protein.

Component	Measured result	Reference range
Color	Pale yellow	-
Clarity	Clear	-
pH	6.5	5-9
Specific gravity	1.015	1.000-1.060
Glucose	Negative	Negative
Blood	Negative	Negative
Ketones	Negative	Negative
Protein	2+	Negative
Urobilinogen	Negative	Negative
Bilirubin	Negative	Negative
Leukocyte esterase	2+	Negative
Nitrite	Negative	Negative

**Figure 1:**
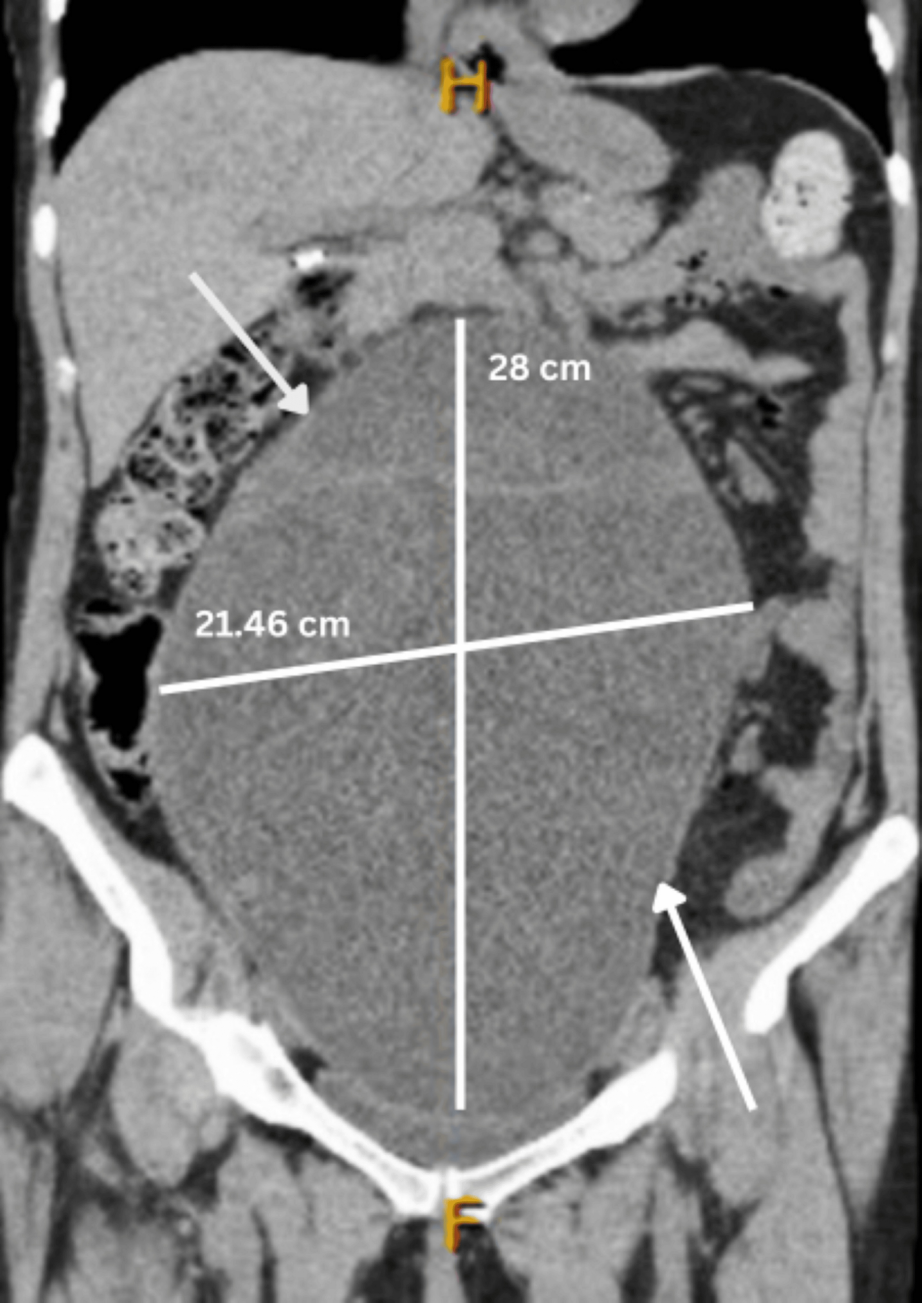
Coronal MRI image of the right borderline ovarian tumor measuring 21.46 cm x 28 cm.

**Figure 2:**
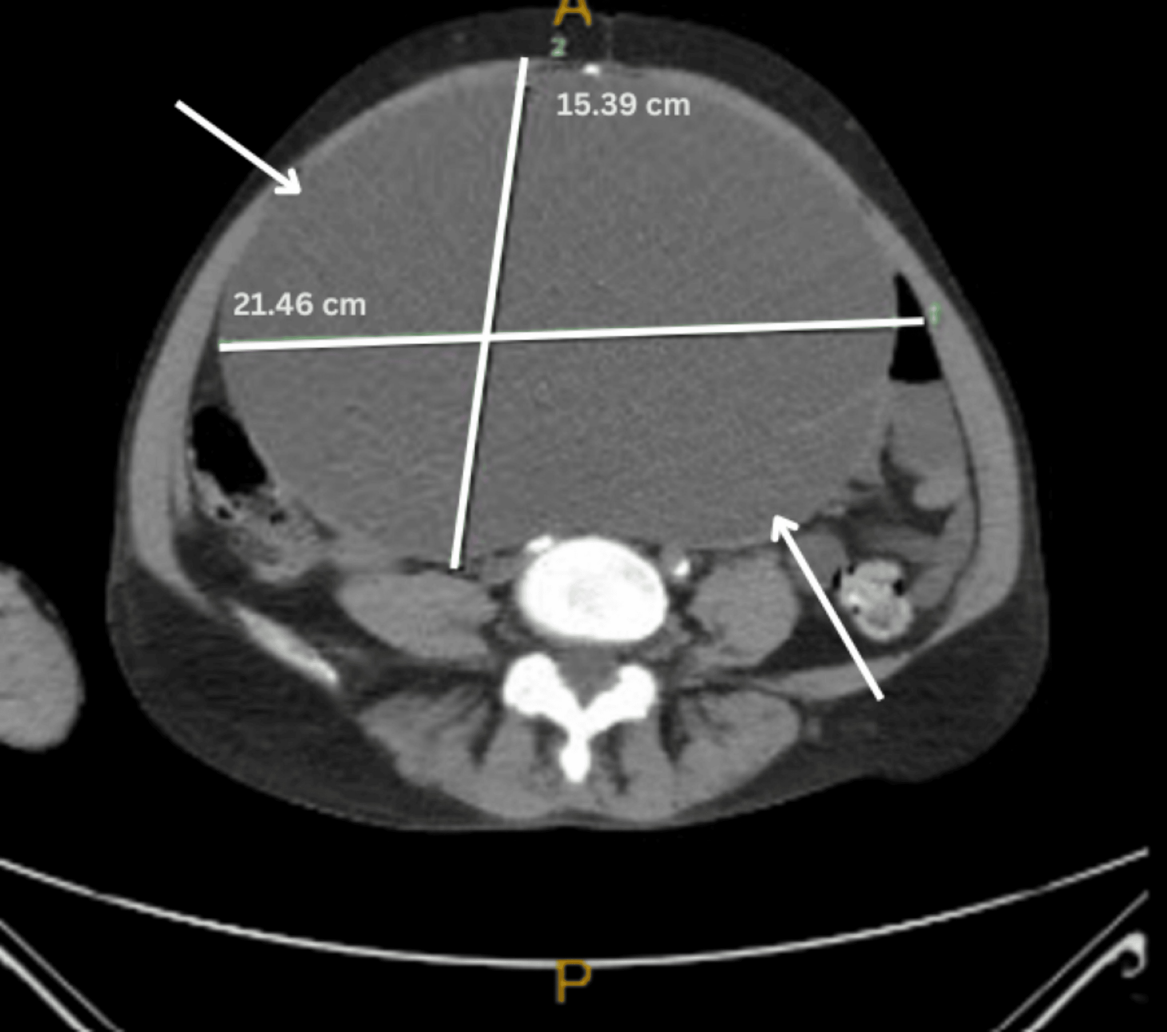
Transverse MRI image of the right borderline ovarian tumor measuring 21.46 cm x 15.39 cm.

**Figure 3:**
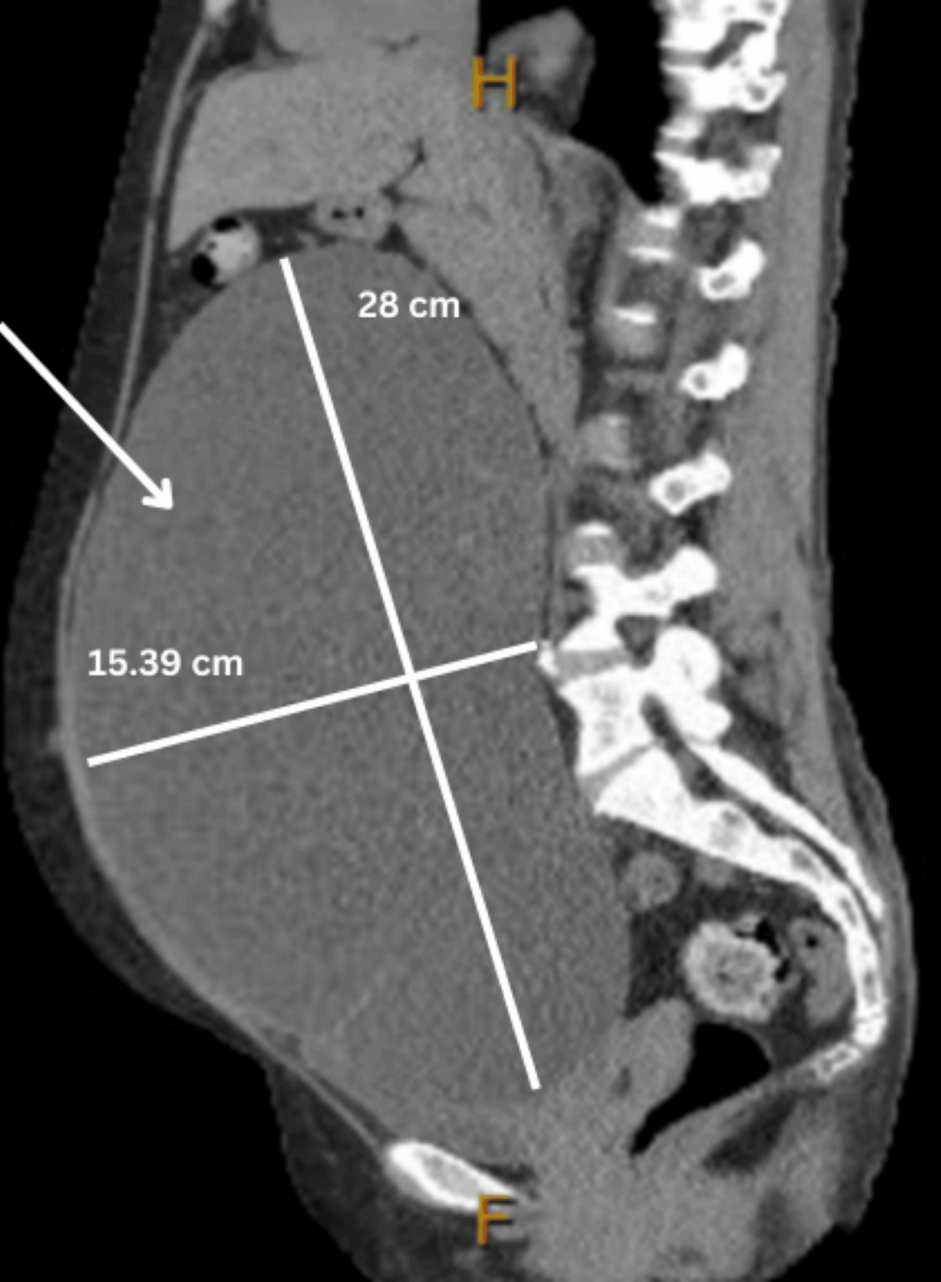
Sagittal MRI image of the right borderline ovarian tumor measuring 28 cm x 15.39 cm.

With an underlying gynecologic malignancy likely, respective cancer markers were ordered. Cancer antigen 125 (CA-125) and carcinoembryonic antigen (CEA) were within normal limits, whereas cancer antigen 19.9 (CA 19.9) was elevated at 736.5 (Table 2). A bedside endometrial biopsy was obtained, and a pathology report indicated the specimen was reported to be “benign endometrium with squamous and endocervical tissue without dysplasia.” A pap smear with HPV co-testing was performed and resulted as negative for intraepithelial lesions or malignancies and negative for HPV. A computed tomography (CT) of the abdomen and pelvis without contrast was performed, which revealed an enormous complex cystic mass arising from the pelvis and extending into the upper abdomen. A significant mass effect was noted. The maximum dimension, superior-to-inferior dimension, was 28 cm, and the radiologist reported that the mass was most likely representing a tumor originating from the right ovary. The transverse dimension of the mass was 21.46 cm, the depth was 15.39 cm, and the vertical dimension was 28 cm (Figures 1-3).

**Table 2:** Associated tumor markers demonstrating normal values for both CA-125 and CEA with an abnormal value for CA 19.9.

Tumor marker	Measured result	Reference range
CA-125	20.1 U/mL	0-35 U/mL
CEA	1.1 ng/mL	0-2.9 ng/mL
CA 19.9	736.5 U/mL	0-37 U/mL

The patient was determined to be a good surgical candidate. After the risks, benefits, and alternatives of an abdominal hysterectomy were explained, informed consent was collected from the patient. The patient underwent a total hysterectomy with bilateral oophorosalpingectomy. A vertical midline abdominal incision from the pubis to above the umbilicus was performed. Pelvic washing was obtained and sent to intraoperative pathology. The mass was dissected off the ureter and inferior pelvic vessels. The large ovarian mass was completely excised and identified as a large right ovarian cyst. In addition, a total hysterectomy was performed since the patient reported that she was complete with childbearing. The case was completed without complications and the incision was closed with staples.

The tissue biopsy obtained during surgery resulted in an ovary with histology consistent with a mucinous BOT measuring 32 cm at its largest diameter. In addition, the fallopian tube had benign epithelium negative for involvement by BOT, and the cervix was negative for malignancy. Perineal washing that was obtained intraoperative also returned as non-malignant cytology. Further investigation of the ovarian histology was conducted, and the results were negative for the following tumor mutations: PIK3CA, KRAS, IDH1, and BRAF. 

The patient met all her postoperative milestones and was discharged on postoperative day three. The patient presented for the follow-up appointment, staples were removed, and no complications were noted. 

## Discussion

This case highlights the clinical presentation, diagnosis, and surgical management of a large mucinous BOT in a 48-year-old woman [2,3]. Mucinous BOTs are rare epithelial ovarian tumors, comprising approximately one-third of BOT cases and 10-15% of all ovarian tumors. Although primarily benign or borderline, these lesions can cause significant symptoms due to their size and anatomical impact, as seen in this case [8,9].

The patient presented with symptoms directly related to the tumor’s size and location, including constipation, abdominal distension, and positional shortness of breath. These mass-related symptoms are characteristic of mucinous BOTs, which most often occur in premenopausal or perimenopausal women. The tumor in this case was particularly large, measuring 32 cm at its greatest dimension, significantly exceeding the average size of mucinous BOTs (20 cm) [10]. The lack of screening tools for early detection underscores the importance of thorough evaluation in women with abdominopelvic symptoms, particularly those without access to routine gynecologic care, as was true for this patient [8,9].

Diagnostic workup included point-of-care ultrasound (POCUS) and computed tomography (CT), which confirmed the presence of a large, complex, septated adnexal mass originating from the ovary. Tumor markers, including CA-125, CEA, and CA 19-9, were assessed, with only CA 19-9 significantly elevated. This result highlights the limited diagnostic sensitivity of tumor markers for BOTs and emphasizes the importance of integrating imaging and histopathological studies into the diagnostic process.

Surgical management in this case was tailored to the patient’s post-reproductive status, consisting of a total hysterectomy with bilateral salpingo-oophorectomy and appendectomy. This approach ensured comprehensive treatment by removing the tumor and eliminating future malignancy risks. Histopathological analysis confirmed the diagnosis of a mucinous BOT without malignant transformation or involvement of surrounding structures. Genetic testing revealed no mutations in common oncogenes, such as KRAS, BRAF, or PIK3CA, supporting the borderline nature of the tumor.

The patient experienced an uncomplicated postoperative recovery, meeting all milestones and achieving favorable outcomes. This case underscores the importance of timely surgical intervention for large ovarian masses. While fertility-sparing surgery is preferred for younger patients, definitive management was appropriate here given the patient’s completed family planning [13].

This case also highlights the critical role of addressing barriers to care. Delayed diagnosis and treatment often stem from limited access to healthcare, as seen in this patient, who had not undergone routine gynecologic evaluations for over a decade. This emphasizes the need for clinicians to maintain a high index of suspicion for ovarian tumors in women with nonspecific symptoms and significant pelvic masses. Early diagnosis and treatment are pivotal, as five-year survival rates for mucinous BOTs exceed 90% when detected early.

Furthermore, this case underscores the systemic importance of improving access to healthcare. Expanding healthcare access, particularly by addressing financial barriers such as lack of insurance coverage, can lead to earlier detection and better management of gynecologic conditions. For instance, the Oregon Health Insurance Experiment demonstrated that Medicaid expansion significantly improved healthcare utilization [14].

Given the absence of effective screening protocols for many gynecologic cancers, expanding access to healthcare could allow for earlier diagnosis, prompt treatment, and improved patient outcomes. Research consistently shows that insured patients are more likely to receive multimodal therapies and care at high-volume centers and experience better survival rates [14]. Addressing these systemic gaps, particularly through expanded insurance coverage, has the potential to significantly improve survival and quality of life for patients with ovarian and other gynecologic tumors.

## Conclusions

This case exemplifies the challenges and complexities associated with the diagnosis and management of mucinous BOTs. The patient’s presentation, with advanced mass-related symptoms, highlights the critical need for thorough evaluation and timely intervention, especially in individuals with limited access to routine gynecological care. Despite the large size of the tumor and its potential complications, surgical management tailored to the patient’s reproductive status ensured an optimal outcome, with complete tumor excision and a favorable recovery.

This case underscores the importance of improving healthcare access to address disparities that delay diagnosis and treatment. Expanding insurance coverage and healthcare availability may enable earlier detection and comprehensive management, ultimately improving survival rates and quality of life for women with gynecologic tumors. Continued efforts to integrate advanced imaging, histopathological evaluation, and patient-centered surgical approaches remain essential in achieving better outcomes for this challenging pathology.
